# Clinical Outcomes of Immediate Versus Staged Revascularization of Nonculprit Arteries in Patients With Acute Coronary Syndrome: A Systematic Review and Meta‐Analysis

**DOI:** 10.1002/clc.70105

**Published:** 2025-03-11

**Authors:** Farah Yasmin, Syeda Farwa Zaidi, Abdul Moeed, Maryam Shahzad, Muhammad Sohaib Asghar, Mahnoor Sadiq, Javed Iqbal, Salim Surani, M. Chadi Alraies

**Affiliations:** ^1^ Yale School of Medicine New Haven Connecticut USA; ^2^ Dow Medical College Karachi Pakistan; ^3^ AdventHealth Sebring Florida USA; ^4^ Nursing Department Hamad Medical Corporation Doha Qatar; ^5^ Texas A&M University College Station Texas USA; ^6^ Detroit Medical Center, Cardiovascular Institute, DMC Heart Hospital Detroit Michigan USA

**Keywords:** immediate revascularization, multivessel disease, percutaneous coronary intervention, staged revascularization

## Abstract

**Background:**

Recent guidelines for acute coronary syndrome (ACS) with multivessel coronary artery disease (MVD) recommend revascularization of non‐culprit lesions following primary percutaneous coronary intervention (PCI). However, the optimal timing for this procedure—whether immediate or staged—remains uncertain.

**Methods:**

A comprehensive search using PubMed (MEDLINE), Cochrane Central, and Google Scholar was conducted to identify studies comparing clinical outcomes between immediate and staged revascularization approaches in patients with MVD undergoing PCI. A random effects model was used to calculate risk ratios (RRs) for dichotomous outcomes with 95% confidence intervals (CIs). The primary outcome was 1‐year all‐cause mortality.

**Results:**

A total of 10 randomized controlled trials (RCTs), comprising 3886 patients (1964 in the immediate revascularization group and 1940 in the staged revascularization group), with a median follow‐up of 12 months, were included in the analysis. No significant difference in the risk of 1‐year mortality was noted between the two approaches. The risk of target vessel revascularization (TVR) at 1‐year follow‐up was significantly lower in the immediate revascularization group compared to the staged revascularization group (RR: 0.64; 95% CI: 0.47–0.86; I²: 0%; *p* = 0.03). Additionally, the immediate revascularization group had a significantly lower risk of myocardial infarction (MI) at 1‐year follow‐up than the staged approach (RR: 0.57; 95% CI: 0.37–0.88; I²: 10%; *p* = 0.01).

**Conclusion:**

This meta‐analysis suggests that immediate revascularization is associated with a significantly lower risk of TVR and MI at 1‐year compared to staged revascularization.

## Graphical abstract

Immediate revascularization is associated with a significantly lower risk of target vessel revascularization and myocardial infarction at 1‐year compared to staged revascularization in patients with multivessel disease undergoing primary percutaneous intervention.

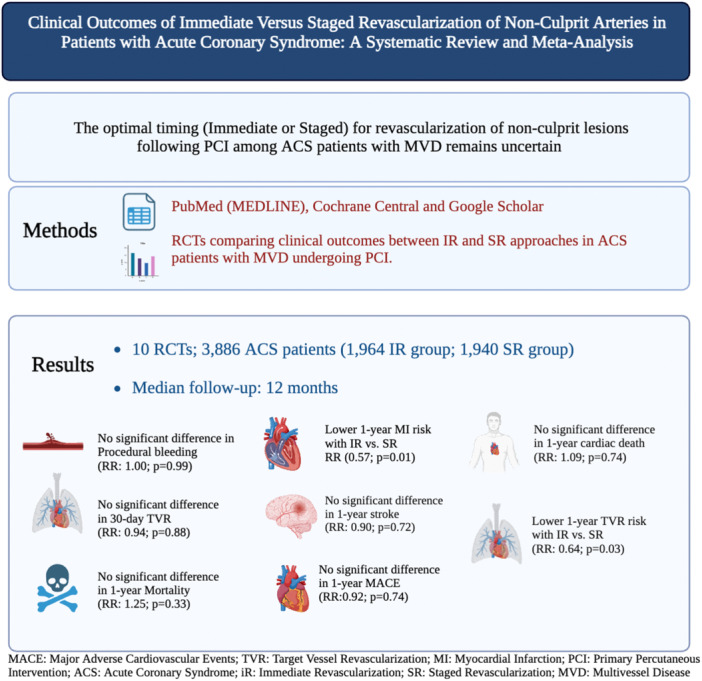

**Abbreviations**
ACS: acute coronary syndrome
BMI: body mass index
CHD: coronary heart disease
CIN: contrast‐induced nephropathy
CK‐MB: creatine kinase‐myocardial band
DES: drug‐eluting stents
IR: immediate revascularization
LAD: left anterior descending
LVEF: left ventricular ejection fraction
MACE: major adverse cardiac events
MVD: multivessel disease
NSTEMI: non‐ST‐elevation myocardial infarction
PAD: peripheral artery disease
PCI: percutaneous coronary intervention
SBP: systolic blood pressure
SR: staged revascularization
STEMI: ST‐elevation myocardial infarction
TVR: target vessel revascularization

## 1 Introduction

Coronary heart disease (CHD) is responsible for approximately 17.8 million deaths worldwide each year [1]. It manifests as an acute coronary syndrome (ACS), including ST‐elevation myocardial infarction (STEMI), non‐ST‐elevation myocardial infarction (NSTEMI), and unstable angina. The American College of Cardiology reports that nearly 50% of the STEMI patients referred for primary percutaneous coronary intervention (PCI) to treat the culprit artery also exhibit multivessel disease (MVD) [2] ACS patients commonly have nonculprit coronary artery disease, which refers to lesions in arteries other than the one causing the ACS. This poses a management challenge and can result in major adverse cardiac events (MACE) if left unaddressed. In contrast, complete multivessel revascularization represents the preferred intervention for treating non‐culprit lesions in ACS patients [3]. The optimal timeline of revascularization remains uncertain. Following treatment of the culprit lesion in ACS patients, complete revascularization can be performed during the index procedure as immediate complete revascularization, or conducted as staged complete revascularization during the index hospitalization or even in ambulatory care.

Emerging as a potential approach to enhance outcomes in ACS patients, immediate complete revascularization aims to address each significant lesion identified during the PCI. This strategy is based on the notion that by treating critical abnormalities in a single intervention, patients can benefit from reduced procedural risks and improved long‐term outcomes. Conversely, evidence suggests that staged complete revascularization may offer a superior approach by minimizing procedural hazards and optimizing long‐term effects [4]. A previous meta‐analysis conducted by Vriesendorp et al. including 10 737 patients determined that the immediate group was associated with a significantly greater risk of mortality in nonrandomized studies [5]. However, this effect was not observed in an analysis of randomized control trials (RCTs) only. Since then, newer trials comparing the two strategies have emerged, including the largest trial to date, BIOVASC (Percutaneous Complete Revascularization Strategies Using Sirolimus Eluting Biodegradable Polymer Coated Stents in Patients Presenting With Acute Coronary Syndrome and Multivessel Disease) trial, comprising a total of 1525 participants [6] and the MULTISTARS AMI (Multivessel Immediate vs*.* Staged Revascularization in Acute Myocardial Infarction) trial [7]. Hence, this meta‐analysis aimed to evaluate the differences in clinical outcomes between the two strategies by pooling recent clinical investigations.

## 2 Methods

This systematic review and meta‐analysis adhere to the established protocols outlined by the Preferred Reporting Items for Systematic Reviews and Meta‐Analyses (PRISMA), Cochrane, and Assessing the Methodological Quality of Systematic Reviews‐2 (AMSTAR‐2) guidelines [8, 9].

### 2.1 Literature Search Strategy

We applied a PRISMA search strategy using Boolean operators and a PICO (patient, intervention, comparison, outcomes) to conduct a comprehensive search across online databases such as MEDLINE, Google Scholar, and Cochrane Central [10]. The Boolean operators “OR” and “AND” were employed to combine synonymous and different keywords/Medical subject headings (MeSH) terms [10, 11]. The terms used in the search strategy included “staged revascularization,” “immediate revascularization,” and “acute coronary syndrome.” We conducted a systematic search to identify all RCTs from the inception of the databases until October 2024. No filters were applied based on language, publication year, author name, or institution/country of publication. We further searched ClinicalTrials.gov and preprints to identify grey literature. A detailed account of the complete search string used for each database can be found in Table S1.

### 2.2 Study Selection and Eligibility Criteria

Following the systematic search, all articles were exported to EndNote X9 Reference Manager (Clarivate Analytics, Philadelphia, Pennsylvania) to remove duplicate records from multiple online databases. Initial screening of the remaining articles based on their titles and abstracts was conducted by two independent investigators (F. Y. and S. F. Z.), considering their relevance to the study population. Subsequently, the full‐text versions of the selected articles were assessed. In cases of inconsistencies, they were resolved through collaborative discussions involving a third investigator (A. M.).

RCTs satisfying the following inclusion criteria were included: (a) studies comparing immediate versus stage approaches of revascularization in patients with ACS and MVD undergoing PCI (b) studies reporting at least one of the clinical outcomes of interest. The obtained records underwent evaluation based on their titles and abstracts, and any records that met one of the following criteria were excluded: case reports, observational studies without a comparison group, reviews, meta‐analyses, lack of relevant outcomes or study questions, and conference abstracts. Two independent investigators (F. Y. and M. S.) checked the full‐text versions of the selected records to ascertain whether they met the inclusion criteria. In cases where there were disagreements, a consensus was reached. Additionally, a manual reference search was conducted on relevant literature to ensure that the study was comprehensive.

### 2.3 Study Outcomes

The primary outcome of interest was all‐cause mortality at 1‐year follow‐up. Secondary outcomes included cardiac death, major adverse cardiovascular events (MACE), stroke, myocardial infarction (MI) and target vessel revascularization (TVR) at 30‐days and 1‐year interval. Procedural outcomes included perioperative bleeding.

### 2.4 Data Extraction

Two investigators (F. Y. and M. S.) independently extracted data from the selected articles using predetermined data collection forms. In addition, we extracted data related to both primary and secondary outcomes. The collected baseline characteristics included age, gender, type of ACS (STEMI/NSTEMI/mixed), cardiac risk factors (such as diabetes mellitus, anterior myocardial infarction (MI), three‐vessel disease, hypertension, hyperlipidemia, current smoking), medical history (such as previous MI, history of angioplasty, peripheral artery disease (PAD), history of heart failure, chronic renal failure), CK‐MB peak, body mass index (BMI), history of bypass surgery, and the culprit artery as the left anterior descending (LAD). Procedural characteristics, such as left ventricular ejection fraction (LVEF), systolic blood pressure (SBP) before PCI, door‐to‐balloon time, contrast‐induced nephropathy, Killip class ≥ 2, heart rate, use of drug‐eluting stents (DES), symptoms‐to‐balloon time, TIMI flow grade before PCI, LVEF ≤ 40%, length of hospital stay, therapy at discharge, radial artery access, use of glycoprotein IIb/IIIa inhibitor, total number of stents, and plasma creatinine before PCI were also collected.

### 2.5 Study Quality Assessment

Two investigators (F. Y. and M. S.) independently conducted the methodological quality assessment of the included RCTs using the Cochrane Risk of Bias Tool (RoB‐2) [12, 13]. Inconsistencies were resolved with the involvement of a third investigator (A. M.). The Cochrane Risk of Bias tool assesses bias in five domains: randomization, deviations from intended variation, missing outcome data, measurement of outcome, and selection of reported results. The trials were scored as high, with some concerns, or low risk of bias in each domain.

### 2.6 Statistical Analysis

The statistical analysis was conducted using Review Manager (RevMan) V.5.3 Cochrane Collaboration, London, United Kingdom. Pooled risk ratios (RRs) and their corresponding 95% confidence intervals (CIs) for each endpoint in the included studies were computed using random‐effects models. A *p* < 0.05 was considered statistically significant. The degree of heterogeneity among the included studies was assessed using the Higgins (I^2^) statistic, where an I^2^ value of < 25% was classified as low, a value between 25% and 75% as moderate, and a value above 75% as high [14]. Publication bias was evaluated through a visual inspection of the funnel plots for outcomes with 10 included studies.

### 2.7 Quality Assessment and Publication Bias

Most of the RCTs were categorized as having a moderate overall risk of bias, primarily due to selection, attrition, and detection biases. A detailed assessment of study quality and risk of bias can be found in the supplementary material (Figures S1 and S2, Table S2). To evaluate publication bias for the outcome of all‐cause mortality at a 1‐year follow‐up, funnel plots were constructed. These plots showed no significant bias, as the studies were symmetrically distributed around the summary effect size (Figure S3).

## 3 Results

**Figure 1:**
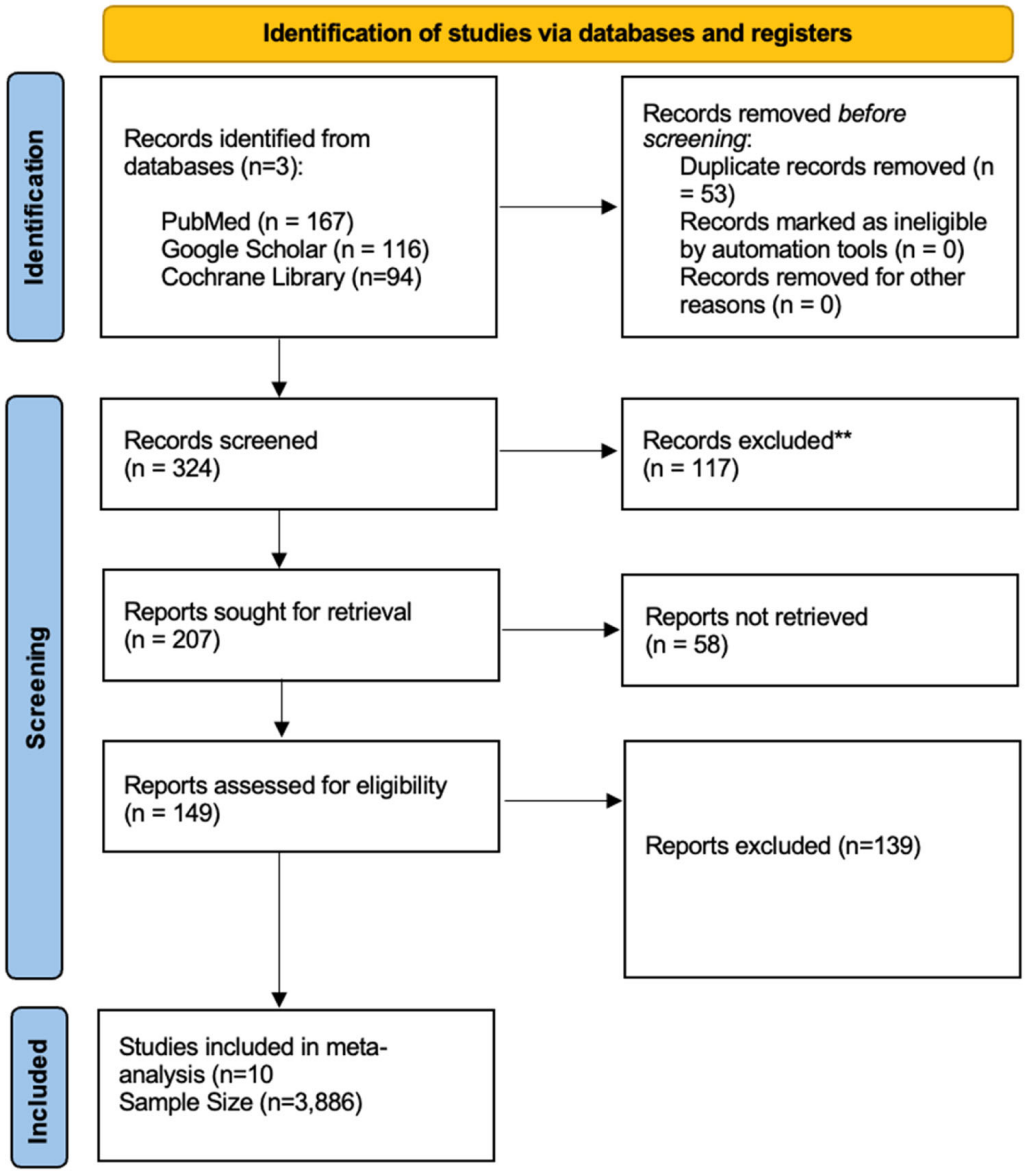
PRISMA flowchart.

An initial search of electronic databases yielded 377 studies. After removing duplicates and applying eligibility criteria, 10 RCTs were included in the meta‐analysis, as illustrated in Figure 1 [6, 7, 15, 16, 17, 18, 19, 20, 21, 22]. This meta‐analysis encompassed 3886 patients with ACS, with 1964 patients in the immediate revascularization (IR) group and 1940 in the staged revascularization (SR) group. The mean age of participants was 63.88 years in the immediate group and 63.49 years in the staged group. The average percentage of male participants was 73.36% in the immediate group and 75.57% in the staged group. Among the included studies, one exclusively studied patients with NSTEMI, another included both NSTEMI and STEMI patients, and the remaining studies focused on STEMI patients. The median follow‐up duration was 12 months (IQR 9–12). Detailed patient characteristics are presented in Table S3.

### 3.1 Outcomes

#### 3.1.1 Periprocedural Outcomes

**Figure 2:**
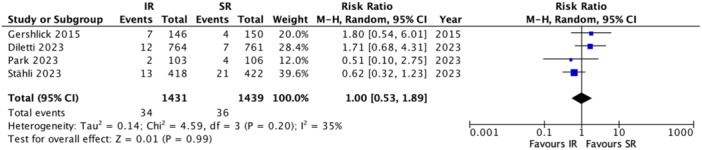
Forest plot of risk of procedural bleeding in immediate versus staged revascularization. CI, 95% confidence interval; RR, risk ratio.

A total of four RCTs reported on the risk of procedural bleeding, totaling 2870 patients (IR: 1431; SR: 1439). Pooled analysis showed no significant difference in the risk of procedural bleeding between IR and SR groups [RR: 1.00; 95% CI: 0.53–1.89; I²: 35%; *p* = 0.99] (Figure 2).

#### 3.1.2 30‐Day Outcomes

**Figure 3:**
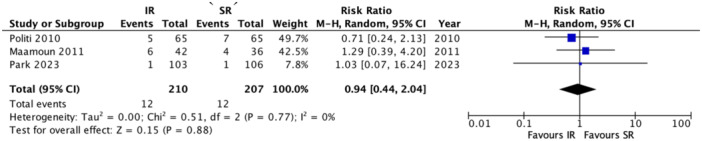
Forest plot of risk target vessel revascularization at 30‐days in immediate versus staged revascularization. CI, 95% confidence interval; RR, risk ratio.

Three RCTs reported on 30‐day target vessel revascularization, including a total of 417 patients (IR: 210; SR: 207). Pooled analysis showed no significant difference in the risk of 30‐day TVR between IR and SR [RR: 0.94; 95% CI: 0.44–2.04; I²: 0%; *p* = 0.88] (Figure 3).

#### 3.1.3 1‐Year Outcomes

**Figure 4:**
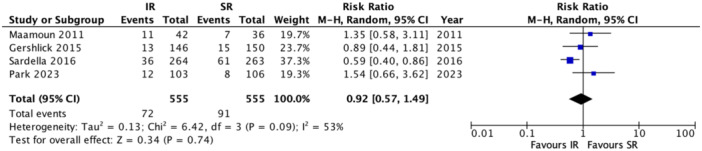
Forest plot for risk of mace (major adverse cardiovascular events) at 1‐year follow‐up in immediate versus staged revascularization. CI, 95% confidence interval; RR, risk ratio.

**Figure 5:**
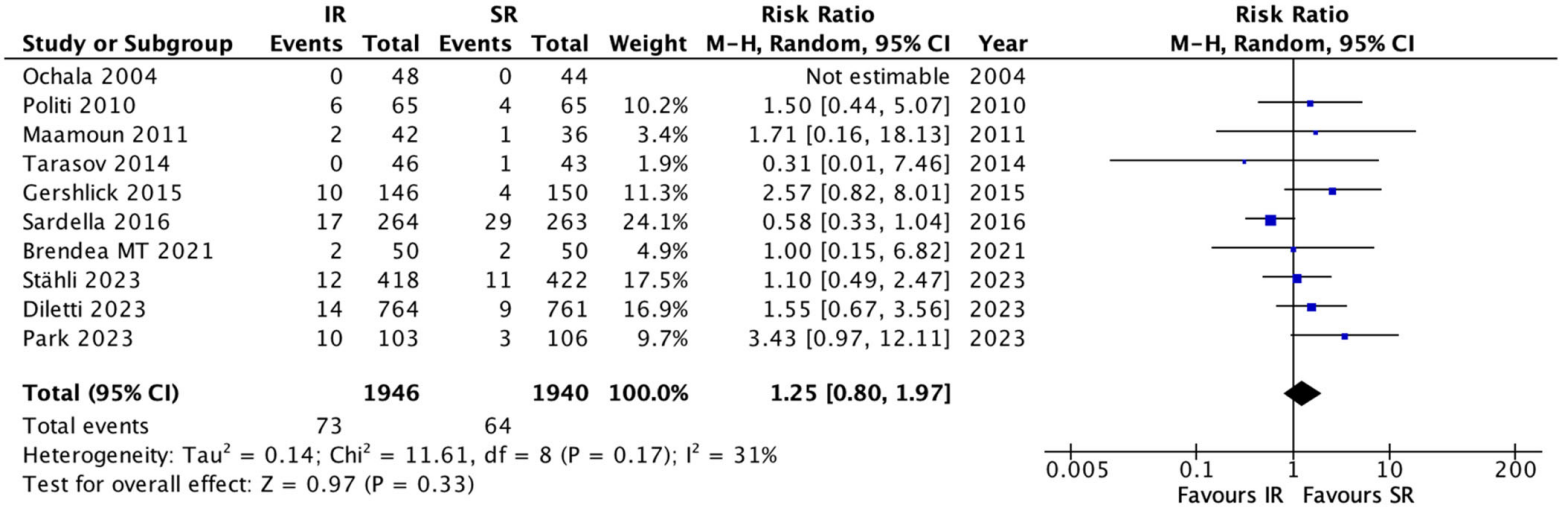
Forest plot of all‐cause mortality at 1‐year follow‐up in immediate versus staged revascularization. CI, 95% confidence interval; RR, risk ratio.

A total of four RCTs reported data on 1‐year major adverse cardiac events (MACE) outcomes, involving 1,110 patients (IR: 555; SR: 555). Pooled analysis indicated no significant difference in the risk of 1‐year MACE between SR and IR [RR: 0.92; 95% CI: 0.57–1.49; I²: 53%; *p* = 0.74] (Figure 4). For 1‐year mortality, data from all 10 RCTs, totaling 3886 patients (IR: 1946; SR: 1940), similarly showed no significant difference in mortality risk between IR and SR [RR: 1.25; 95% CI: 0.80–1.97; I²: 31%; *p* = 0.33] (Figure 5). In contrast, eight RCTs examining 1‐year myocardial infarction (MI) data in 3,656 patients (IR: 1831; SR: 1825) indicated a significantly lower MI risk with IR compared to SR [RR: 0.57; 95% CI: 0.37–0.88; I²: 10%; *p* = 0.01] (Figure S4). Five RCTs, encompassing 3179 patients (IR: 1591; SR: 1588), provided data on 1‐year stroke, revealing no significant difference in stroke risk between IR and SR [RR: 0.90; 95% CI: 0.49–1.64; I²: 0%; *p* = 0.72] (Figure S5). Similarly, for cardiac death at 1‐year follow‐up, five RCTs with a total of 3231 patients (IR: 1614; SR: 1617) showed no significant difference between the two approaches [RR: 1.09; 95% CI: 0.67–1.77; I²: 0%; *p* = 0.74] (Figure S6). Finally, 1‐year TVR data, reported in five RCTs with 3179 patients (IR: 1591; SR: 1588), demonstrated a significantly lower risk of TVR with IR compared to SR [RR: 0.64; 95% CI: 0.47–0.86; I²: 0%; *p* = 0.03] (Figure S7).

### 3.2 Meta‐Regression

Meta‐regression was performed to determine a correlation between the primary outcome, 1‐year mortality, and various baseline covariates (Figures S8–S13). Diabetes mellitus (Coeff; −0.0143, *p* = 0.542), anterior MI (Coeff; −0.007, *p* = 0.862), three‐vessel disease (Coeff; −0.04, *p* = 0.405) and hyperlipidemia (Coeff; −0.026, *p* = 0.237) were not found to be correlated with 1‐year mortality. However, hypertension (Coeff; −0.045, *p* = 0.0034), and a history of angioplasty (Coeff; −0.092, *p* = 0.006) was found to be significantly associated with decreased 1‐year mortality in the IR group.

## 4 Discussion

This systematic review and meta‐analysis including a total of 3886 ACS patients—1964 in the IR group and 1940 in the SR group showed that IR significantly reduce the risk of TVR and MI at 1‐year follow‐up. Outcomes, including 30‐day TVR, and 1‐year rates of MACE, stroke, all‐cause mortality, and cardiac death, in addition to the periprocedural bleeding risk were similar between the two approaches.

Contrary to our findings, the results of two previous meta‐analyses concluded staged procedure, compared with immediate, to be associated with lower risk of mortality [23, 24]. However, it is important to note that these meta‐analyses also included observational studies. Further, our findings are consistent with those of the BIOVASC trial which also revealed no significant differences in 1‐year mortality between IR and SR approaches [6]. However, future RCTs with larger sample sizes are needed to provide more definitive evidence regarding the relative efficacy of these strategies.

A separate meta‐analysis by Hu et al. examined immediate and staged multivessel PCI strategies specifically in STEMI patients [25]. Their findings showed that immediate revascularization was significantly associated with increased rates of short‐ and long‐term mortality, short‐term MACE, and short‐term cardiac death compared to the staged approach. In contrast, our findings suggest that immediate revascularization was associated with reduced incidence of MI at 1‐year, while all other outcomes—including long‐term mortality and cardiac death—were comparable between the two groups [25]. The differences in findings might stem from methodological factors, such as the inclusion of only RCTs in our study and the broader patient population including both STEMI and NSTEMI patients. Hu and colleagues study focused solely on STEMI patients, and their results may be influenced by the specific characteristics of that patient group. Patients in the acute phase of STEMI often experience significant hemodynamic instability and left ventricular dysfunction, which can increase the risk of complications during PCI. The heightened prothrombotic and pro‐inflammatory states in these patients make the entire region more vulnerable to instability, raising the likelihood of procedural issues such as stent thrombosis and abrupt vessel closure [26, 27, 28]. Additionally, the immediate multivessel revascularization approach necessitates higher contrast use, which increases the risk of CIN, a complication that is particularly poorly tolerated in STEMI patients. Assali et al. observed a stepwise increase in 30‐day mortality in acute MI patients with varying degrees of renal impairment, with the highest mortality seen in those with severe renal dysfunction (*p* = 0.004) [27]. Marenzi and colleagues also found a higher in‐hospital mortality rate among STEMI patients who developed CIN after PCI (*p* < 0.001) [29].

Additionally, in STEMI, diffuse coronary vasoconstriction and pronounced endothelial dysfunction may lead to overestimation of stenosis in nonculprit vessels, further complicating the decision for immediate multivessel intervention. Moreover, complications arising from an IR strategy may be particularly difficult to manage due to the “double jeopardy” of handling both the culprit and nonculprit regions simultaneously [29]. In contrast, for NSTEMI patients, the results are more mixed. Subgroup analysis of intermediate to high‐risk NSTEMI patients suggests that the staged approach may reduce the risk of short‐term MACE and cardiac death compared to the immediate approach (*p* = 0.037 and *p* = 0.031, respectively) [30].

Our analysis demonstrated that the immediate revascularization group had significantly fewer MI events at 1‐year follow‐up compared to the staged group. In the context of ACS, nonculprit lesions may harbor unstable plaques that are at risk of rupture. Goldstein and colleagues found that the presence of multiple complex plaques was associated with an increased risk of recurrent ACS (*p* < 0.001) [31]. Additionally, in the BIOVASC trial, most MI events in the staged group occurred shortly after the index procedure, rather than during or after the staged intervention [6]. Furthermore, nonculprit lesions were responsible for most of these MI events. This suggests that the risk of MI may be higher during the interval between the index and staged procedures, particularly in patients with more severe and complex nonculprit lesions. Therefore, these patients may benefit more from IR, which could explain why our study found fewer MI events at 1‐year follow‐up in the immediate revascularization group.

The limitations of this meta‐analysis require careful consideration. Although the sample size was substantial, the relatively small number of included RCTs may impact the generalizability of the findings, as these trials may not fully capture the diversity of patient populations and procedural settings. Additionally, variability in study protocols, including differences in baseline characteristics, could introduce heterogeneity, affecting the consistency of the results. Inconsistent definitions and reporting of adverse events across studies may further limit comparability of the assessed outcomes. Another significant limitation is the lack of long‐term follow‐up data in most studies, which restricts our ability to extrapolate the long‐term effects of immediate versus staged revascularization, especially in high‐risk populations, such as the elderly, individuals with renal impairment, diabetes, hypertension, hyperlipidemia, or a history of MI. Furthermore, the RCTs included a greater proportion of male participants than female, which may limit the extrapolation of these findings to the female population. Moreover, the choice of revascularization strategy was determined at the discretion of the operator, which means that potential confounders—such as patient characteristics and operator experience—could not be fully adjusted for in the analysis.

## 5 Conclusion

This meta‐analysis included recently published data from the BIOVASC and MULTI STARS AMI trials, and provided a comprehensive evaluation of the clinical outcomes of IR versus SR in ACS patients with MVD. The findings demonstrated IR to be associated with lower risk of TVR and MI at 1‐year follow‐up compared to the staged approach.

## Author Contributions

Farah Yasmin, Abdul Moeed, Syeda Farwa Zaidi, and Maryam Shahzad participated in the conceptualization, data curation, and writing of the original draft. Mahnoor Sadiq and Muhammad Sohaib Asghar participated in the investigation, methodology, project administration, resources, and writing of the original draft. M Chadi Alraies and Salim Surani participated in the supervision, validation, visualization, and writing, review, and editing.

## Ethics Statement

The authors have nothing to report.

## Consent

The authors have nothing to report.

## Conflicts of Interest

The authors declare no conflicts of interest.

## Supporting information

Supporting information.

## Data Availability

The data that support the findings of this study are available from the corresponding author upon reasonable request.
