# Supplementary material for: Clinical Outcomes of Immediate Versus Staged Revascularization of Nonculprit Arteries in Patients With Acute Coronary Syndrome: A Systematic Review and Meta‐Analysis
Source: Clin Cardiol. 2025 Mar 11;48(3):e70105. doi: 10.1002/clc.70105 (PMC11894271; doi:10.1002/clc.70105)
Supplement: Supplementary file 1 — Supporting information. [file CLC-48-e70105-s001.docx]

**SUPPLEMENTARY FILE**

**Clinical Outcomes of Immediate Versus Staged Revascularization of Non-Culprit Arteries in Patients with Acute Coronary Syndrome: A Systematic Review and Meta-Analysis**

**Table S1. Detailed search strategy in used in each database.**

| **Databases** | **Detailed search strategy** |
| --- | --- |
| PubMed | (staged revasc* OR stage revasc*) AND (immediate revasc* OR non-culprit) AND ("acute coronary syndrome" OR "multivessel disease" OR "ACS" OR "myocardial infarction" OR "unstable angina" OR MVD) |
| Cochrane Central | (staged revasc* OR stage revasc*) AND (immediate revasc* OR non-culprit) AND ("acute coronary syndrome" OR "multivessel disease" OR "ACS" OR "myocardial infarction" OR "unstable angina" OR MVD) |
| Google Scholar | staged revasc* or stage revasc* and immediate revasc* or non-culprit and "acute coronary syndrome" or "multivessel disease" or "ACS" or "myocardial infarction" or "unstable angina" or “MVD’ |

**Table S2: Risk of bias summary of included trials based on Jaded scale**

| **Studies** | **Randomization** | **Blinding** | **Account of all patients** | **Overall score** | **Quality** |
| --- | --- | --- | --- | --- | --- |
| **Gershlick, et al.** | 1 | 0 | 1 | 2/5 | Moderate |
| **Ochala, et al.** | 1 | 0 | 0 | 1/5 | Low |
| **Politi, et al.** | 2 | 1 | 1 | 4/5 | High |
| **Maamoun, et al.** | 2 | 0 | 0 | 2/5 | Moderate |
| **Tarasov, et al.** | 2 | 0 | 2 | 4/5 | High |
| **Sthäli, et al.** | 2 | 1 | 2 | 5/5 | High |
| **Sardella, et al.** | 2 | 0 | 2 | 4/5 | High |
| **Diletti, et al.** | 2 | 0 | 2 | 4/5 | High |
| **Brendea MT, et al.** | 1 | 1 | 2 | 4/5 | High |
| **Park, et al.** | 2 | 0 | 2 | 4/5 | High |

| **Study** | **Design** | **Patient Population** | **Patients, n** | | **Age, years** | | **Men, n (%)** | | **Diabetes mellitus, n(%)** | | **Hypertension, n(%)** | | **Hyperlipidemia, n(%)** | | **History of MI^f^, n(%)** | |
| --- | --- | --- | --- | --- | --- | --- | --- | --- | --- | --- | --- | --- | --- | --- | --- | --- |
|  |  |  | **IR^a^** | **SR^b^** | **IR** | **SR** | **IR** | **SR** | **IR** | **SR** | **IR** | **SR** | **IR** | **SR** | **IR** | **SR** |
|  |  |  |  | |  | |  | |  | |  | |  | |  | |
| **PRIMA, 2004** (17) | RCT^c^ | STEMI^d^ | 48 | 44 | 65 ±8.3 | 67±7.9 | 35(72.9) | 33(75) | 15 (31.2) | 15 (34.1) | 25 (52.1) | 21(47.7) | 39 (81.2) | 40 (90.9) | 14 (29.1) | 10 (22.7) |
| **POLITI, 2010** (15) | RCT | STEMI | 65 | 65 | 64.5 ±11.7 | 64.1±11.1 | 15 (23.07) | 13 (20) | 9 (13.8) | 12 (18.5) | 32 (49.2) | 42 (64.6) | - | - | - | - |
| **MAAMOUN, 2011** (22) | RCT | STEMI | 42 | 36 | 54.52 ± 10.3 | 52.33 ± 7.1 | 2 (4.17) | 4 (11.11) | 17 (40.5) | 20 (55.6) | 16 (38.1) | 12 (33.3) | 24 (57.1) | 16 (44.4) | - | - |
| **TARASOV, 2014** (21) | RCT | STEMI | 46 | 43 | 58,6±11 | 58.9±10.4 | 32 (69.6) | 25 (58.1) | 12 (26.1) | 9 (20.9) | 44 (95.6) | 37 (86) | - | - | 5 (10.8) | 2 (4.6) |
| **SARDELLA, 2016** (16) | RCT | NSTEMI^e^ | 264 | 263 | 72 (61−78)* | 73 (62−78) * | 207 (78.40) | 209 (79.46) | 90 (34.09) | 93 (35.36) | 193 (73.10) | 174 (66.15) | 152 (57.57) | 143 (54.37) | 71 (26.89) | 62 (23.57) |
| **BIOVASC, 2023** (6) | RCT | STEMI and NSTEMI | 764 | 761 | 65·7 (57·2–72·9) * | 65·3 (58·6–72·9) * | 598 (78·3) | 589 (77·4) | 158 (20·7) | 163 (21·4) | 423 (55·4) | 395 (51·9) | 385 (50·5) | 399 (52·5) | 69 (9) | 89 (11·7) |
| **COCUA, 2023** (18) | RCT | STEMI | 103 | 106 | 63.3 ± 10.4 | 62.2 ± 10.9 | 82 (79.6) | 88 (83.0) | 42 (40.7) | 37 (34.9) | 56 (54.3) | 48 (45.2) | 38 (36.8) | 41 (38.6) | 1 (0.9) | 1 (0.9) |
| **CVLPRIT, 2015** (19) | RCT | STEMI | 146 | 150 | 65.3 ± 11.9 | 64.6 ± 11.2 | 112 (76.7) | 128 (85.3) | 20 (14.3) | 19 (12.9) | 51 (36.4) | 54 (36.6) | 34 (24.3) | 41 (27.9) | 5 (3.6) | 7 (4.8) |
| **NICHITA-BRENDEA, 2021** (20) | RCT | STEMI | 50 | 50 | - | - | 37 (74) | 36 (72) | 12 (24) | 11 (22) | 20 (40) | 24 (48) | - | - | - | - |
| **MULTISTARS AMI, 2023 7**) | RCT | STEMI | 418 | 422 | 66 (58–74) | 64 (55–73) | 321 (76.8) | 341 (80.8) | 66 (15.8) | 65 (15.4) | 228 (54.5) | 212 (50.2) | 112 (26.8) | 114 (27.1) | 28 (6.7) | 20 (4.8) |

**Table S3. General characteristics of the included studies.**

*Values are in form of Median (IQR)

^a^Immediate revascularization, ^b^Staged revascularization, ^c^Randomized controlled trial, ^d^ST-elevation myocardial infarction, ^e^non ST-elevation myocardial infarction, ^f^ Myocardial infractio

**Figure S1: Traffic light plot for RoB 2 assessment.**

**
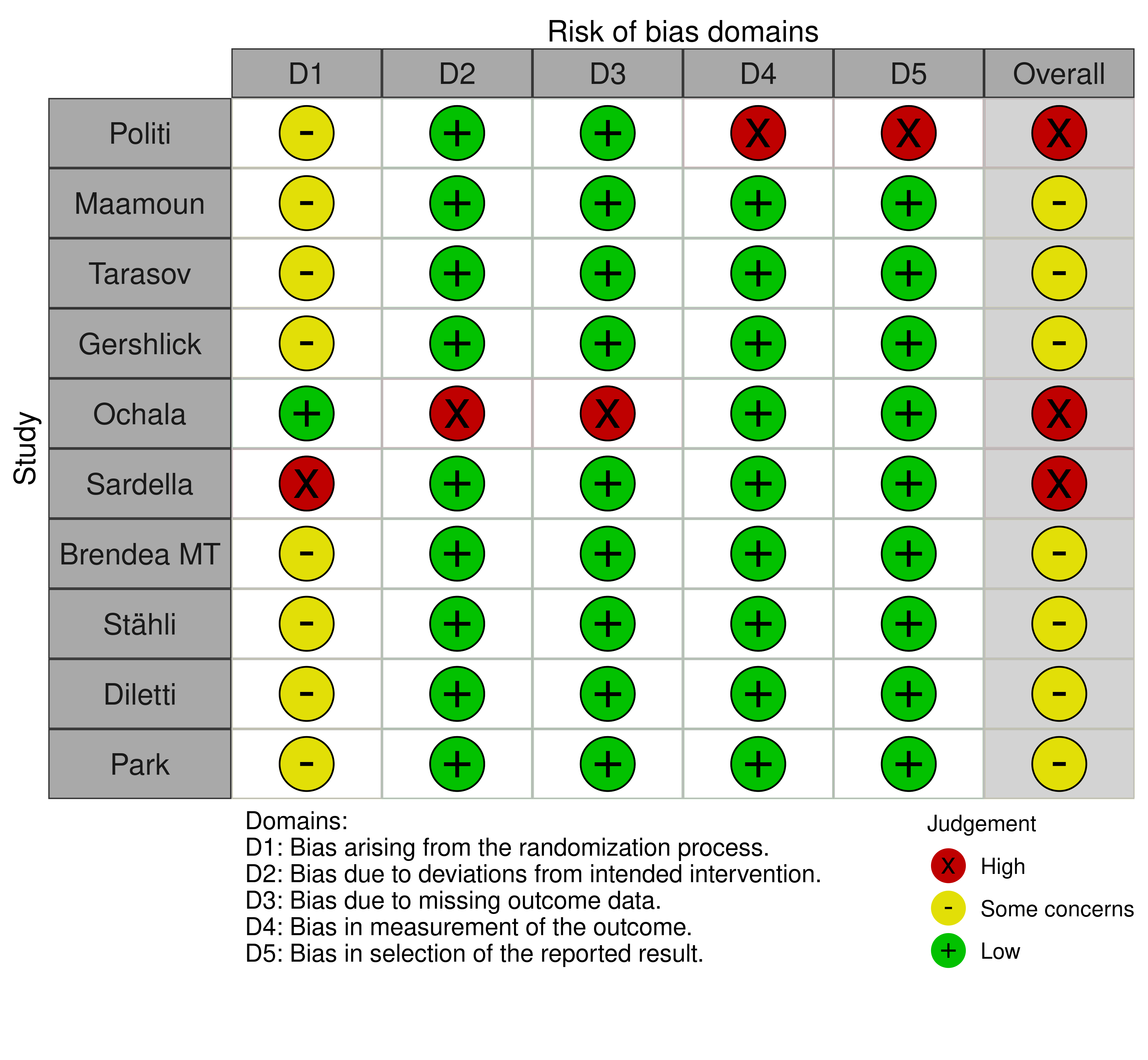
**

**Figure S2: Summary plot for RoB 2 assessment.**

**
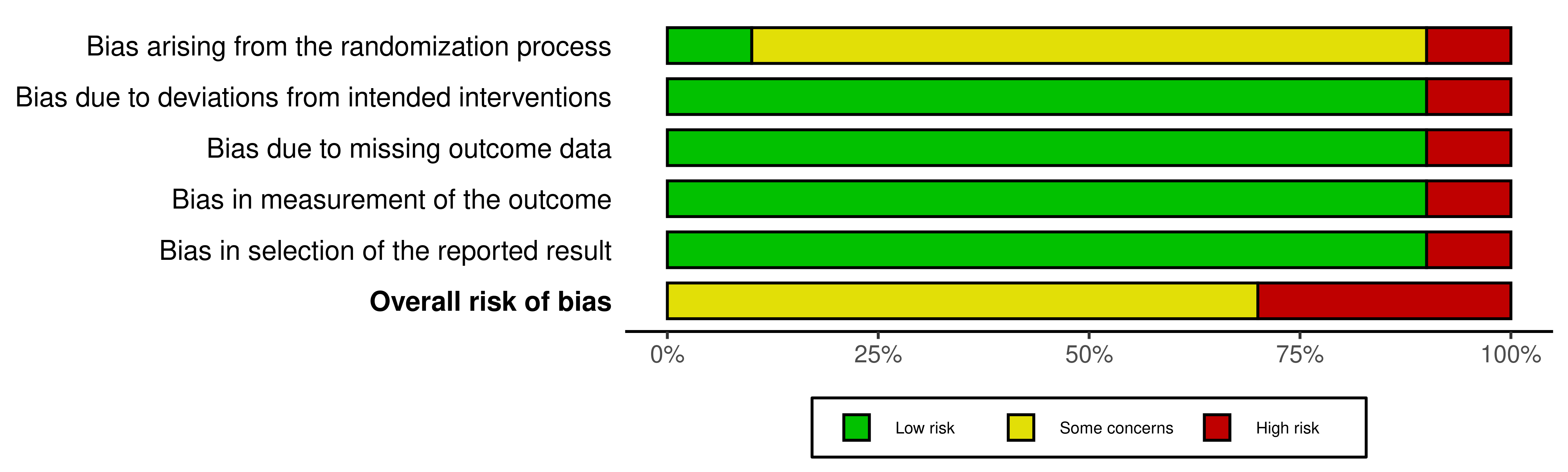
**

**Figure S3: Funnel plot for the outcome of all-cause mortality at 1-year follow-up.**


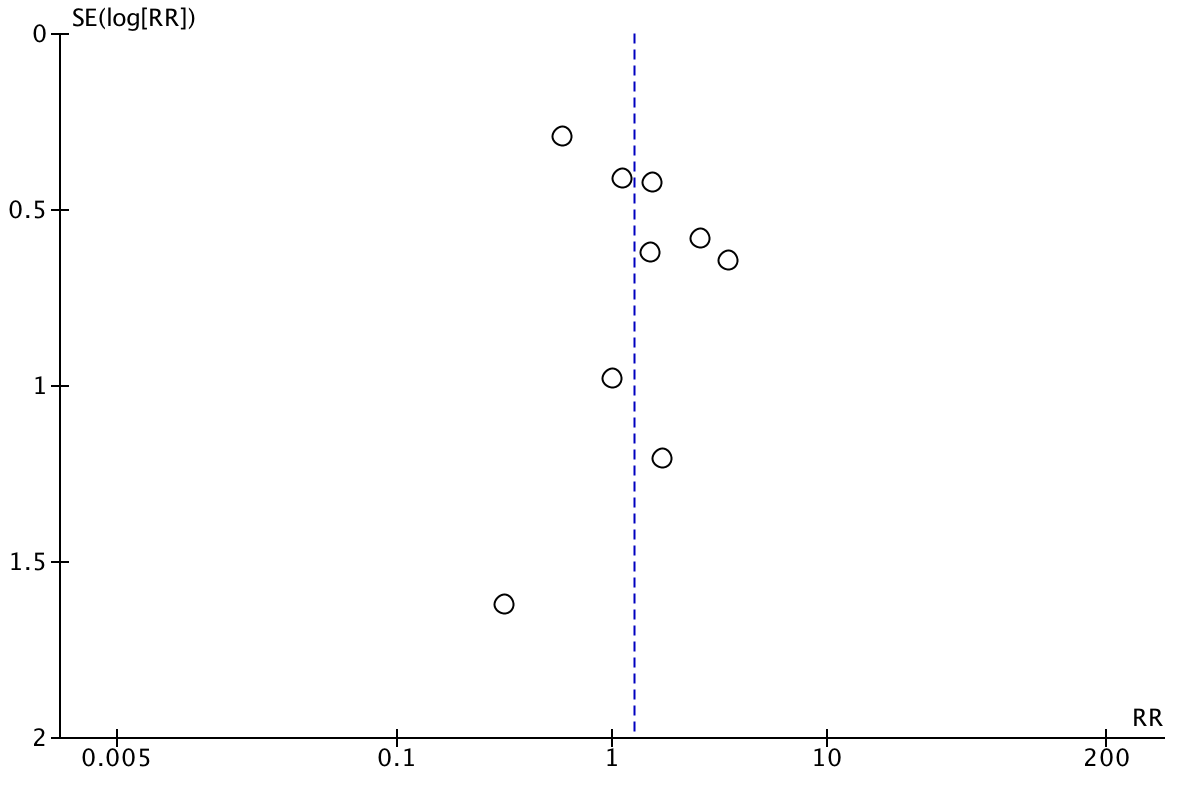


**Figure S4: Forest Plot for risk of Stroke at 1-Year Follow-Up in Immediate vs. Staged Revascularization. Risk Ratio (RR), 95% Confidence Interval (CI).**

**
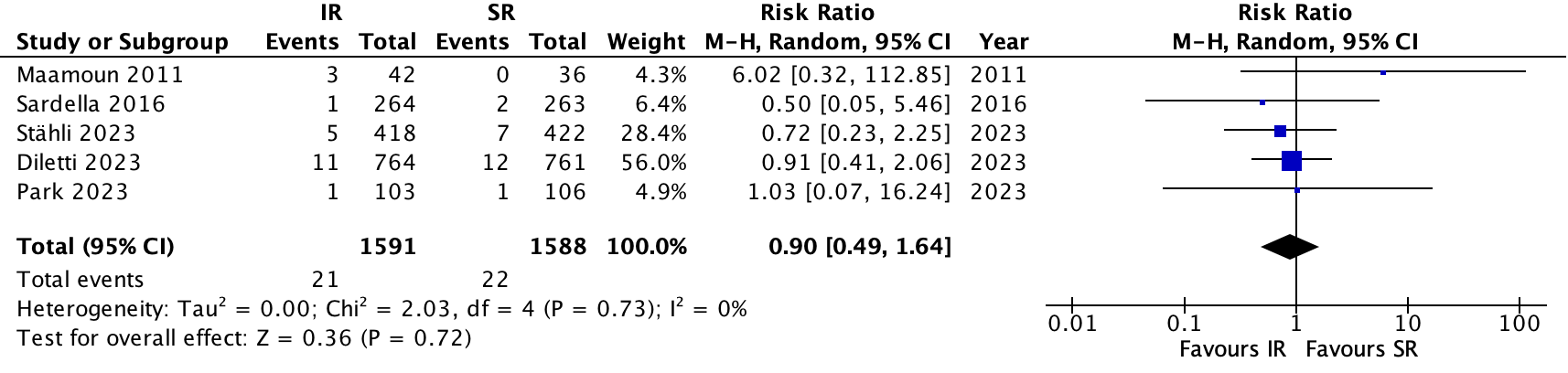
**

**Figure S5: Forest Plot for risk of Myocardial Infarction (MI) at 1-Year Follow-Up in Immediate vs. Staged Revascularization. Risk Ratio (RR), 95% Confidence Interval (CI).**

**
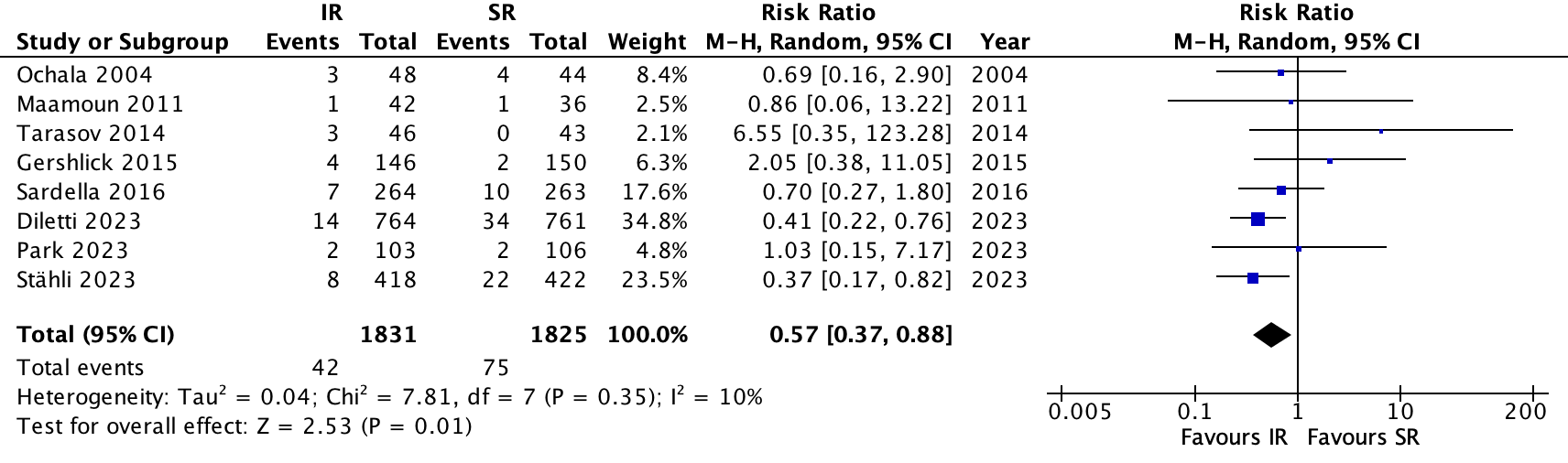
**

**Figure S6: Forest Plot risk of Cardiac Death at 1-Year Follow-Up in Immediate vs. Staged Revascularization. Risk Ratio (RR), 95% Confidence Interval (CI).**

**
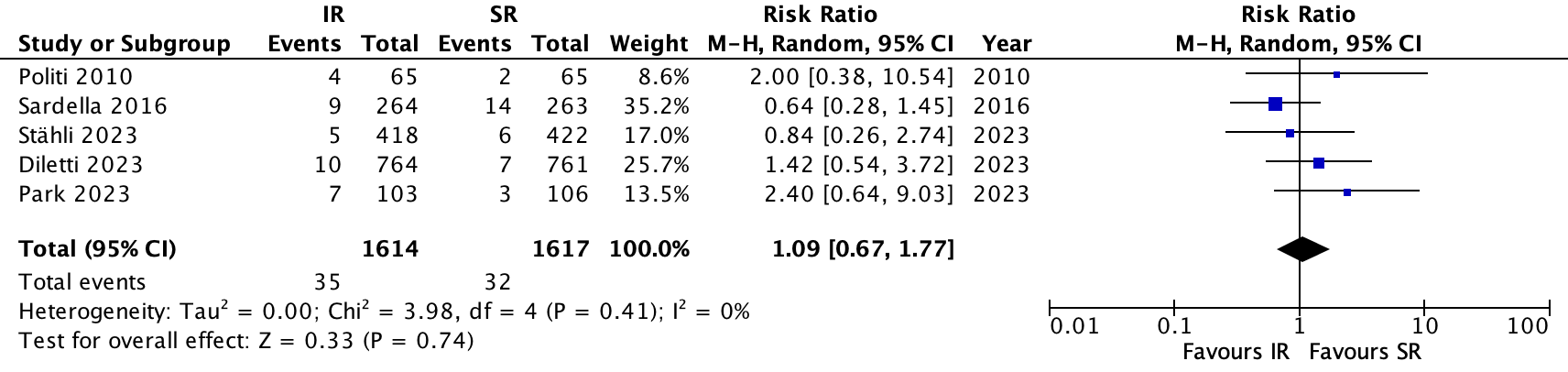
**

**Figure S7: Forest Plot for Risk Target Vessel Revascularization at 1-Year Follow-Up in Immediate vs. Staged Revascularization. Risk Ratio (RR), 95% Confidence Interval (CI).**

**
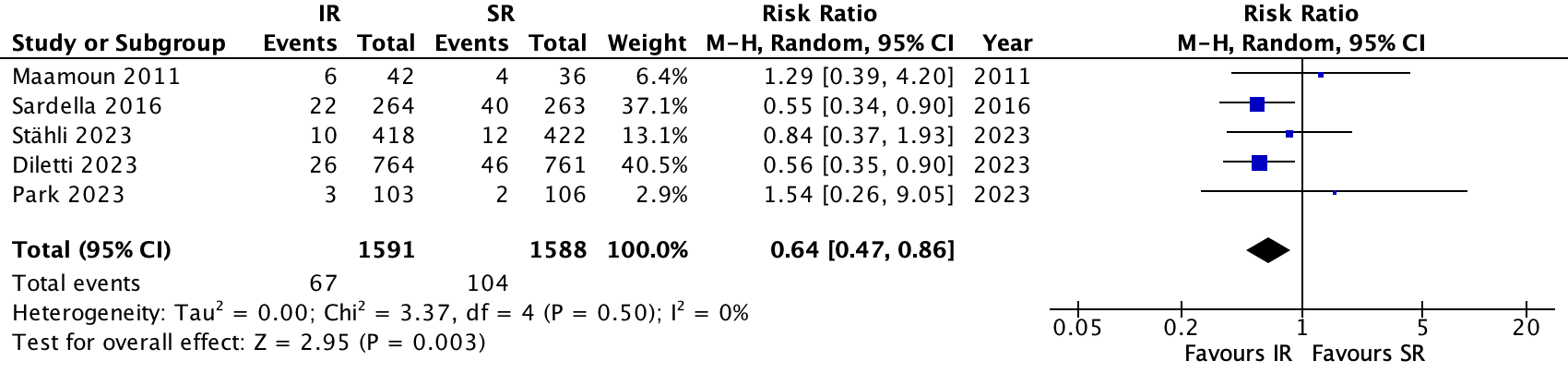
**

**Figure S8. Meta-regression of 1-year mortality and diabetes** (Coeff; -0.0143, p=0.542)

**
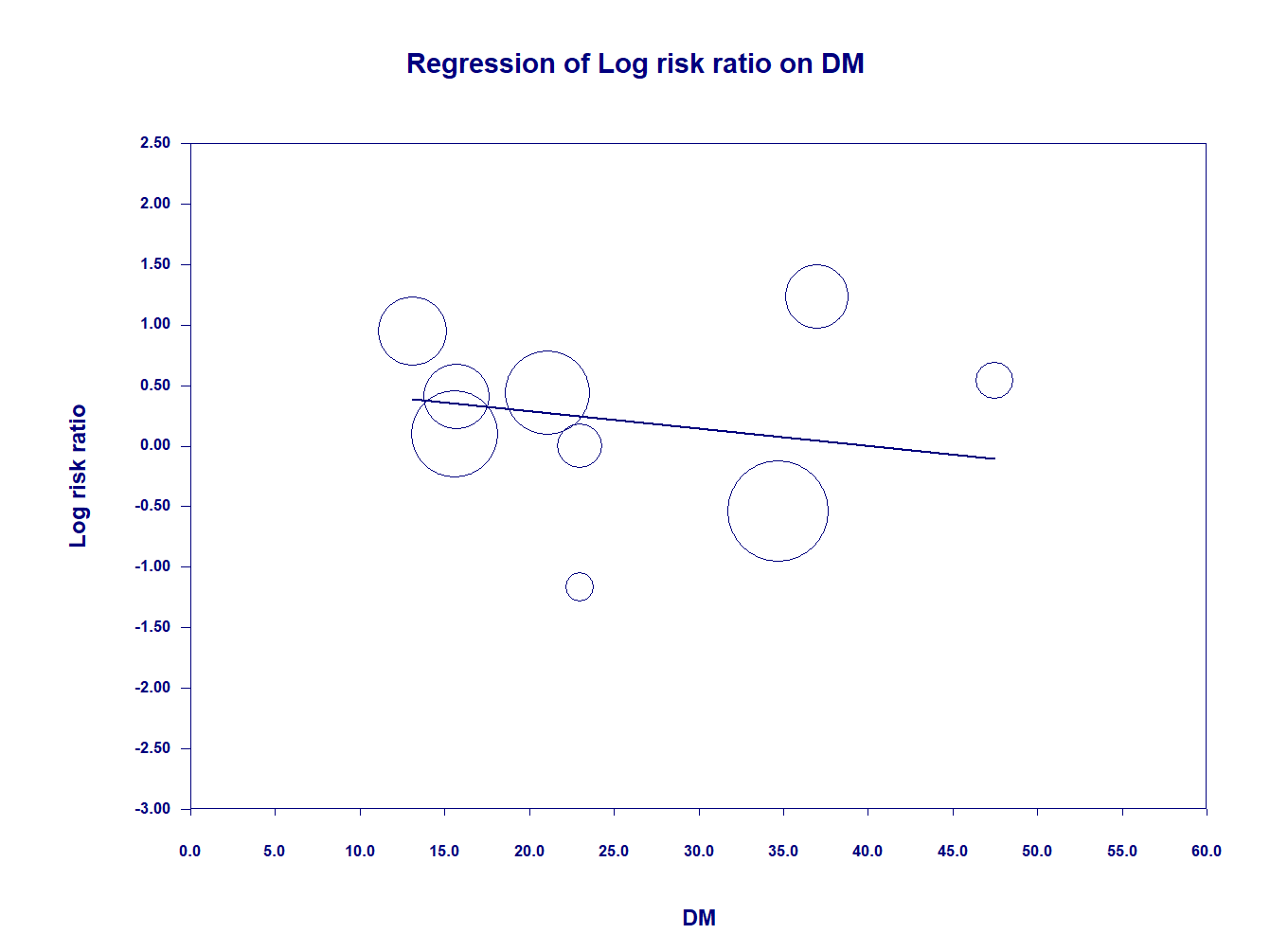
**

**Figure S9. Meta-regression of 1-year mortality and anterior myocardial infarction** (Coeff; -0.007, p=0.862)

**
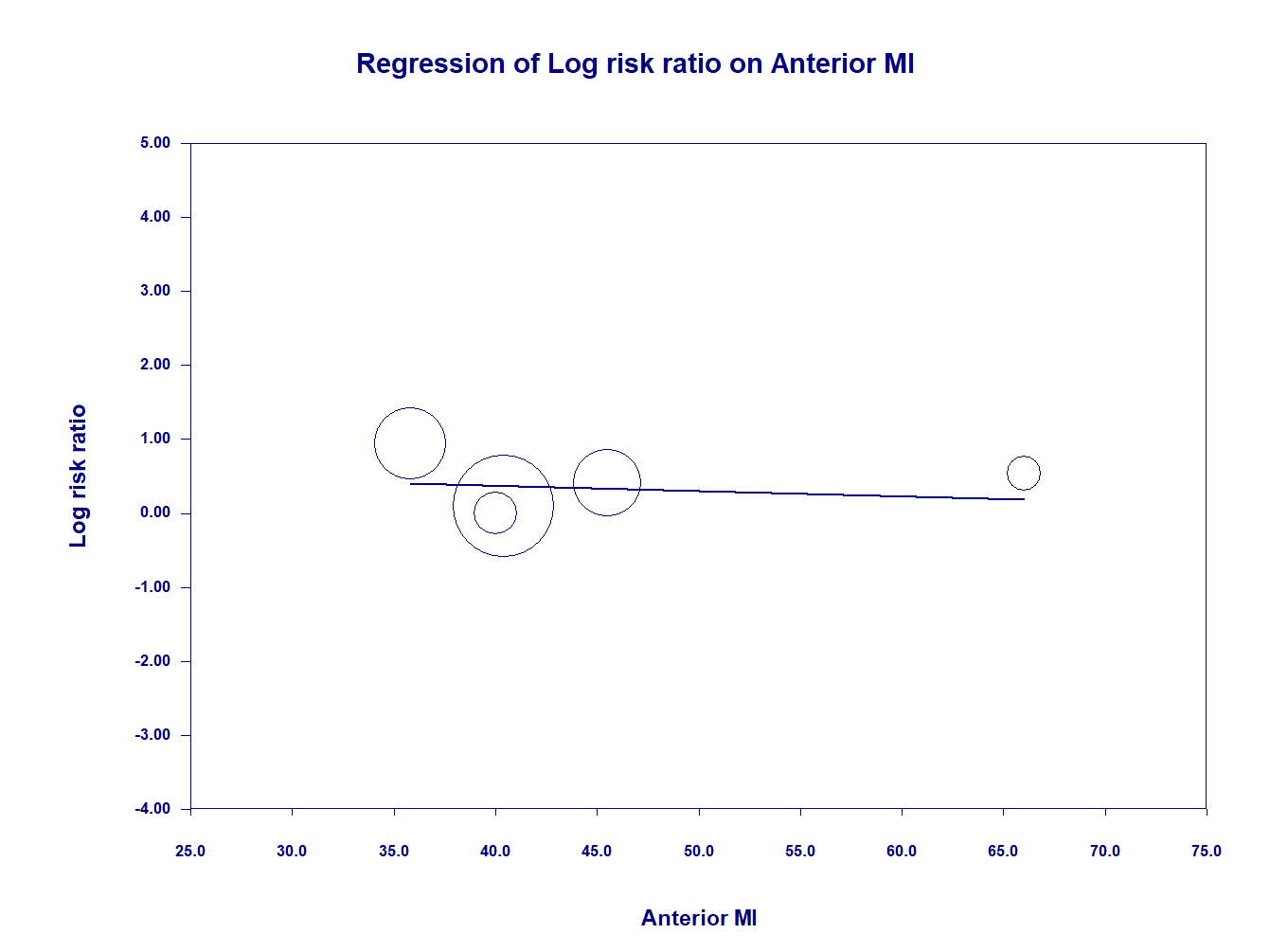
**

**Figure S10. Meta-regression of 1-year mortality and 3-vessel disease (**Coeff; -0.04, p=0.405)**
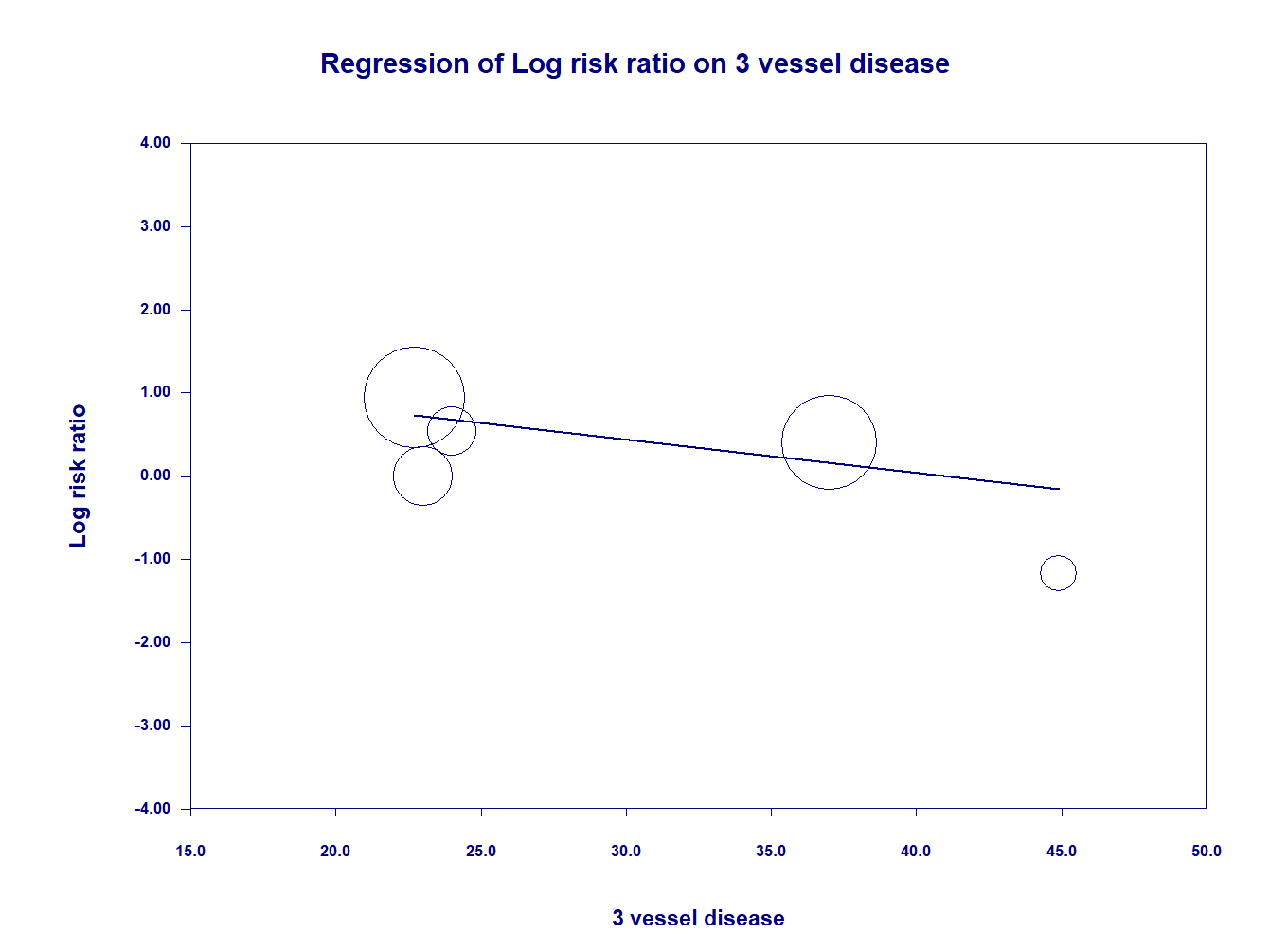
**

**Figure S11. Meta-regression of 1-year mortality and hyperlipidemia** (Coeff; -0.026, p=0.237) **
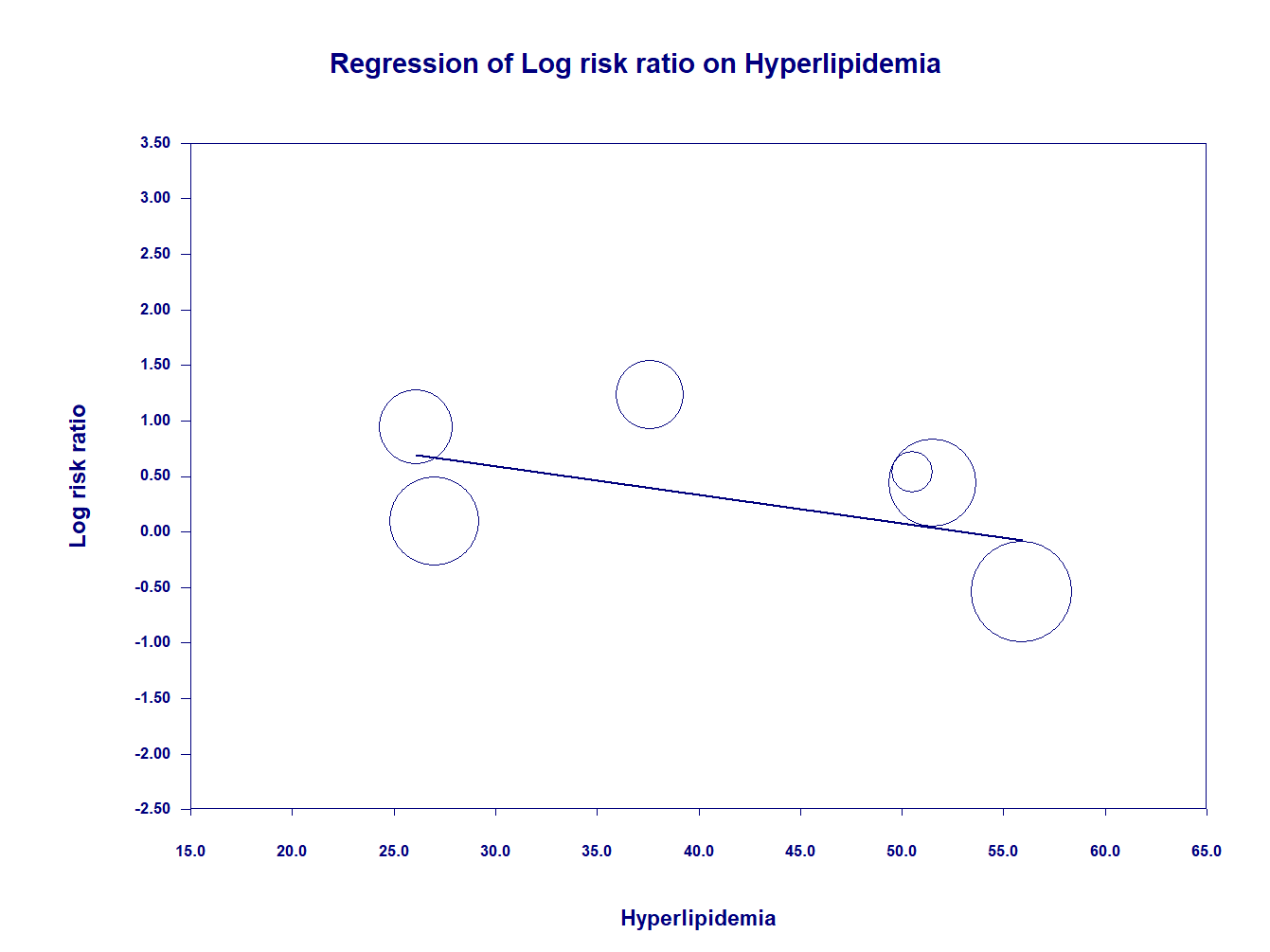
**

**Figure S12. Meta-regression of 1-year mortality and history of angioplasty** (Coeff; -0.092, p=0.006)

**
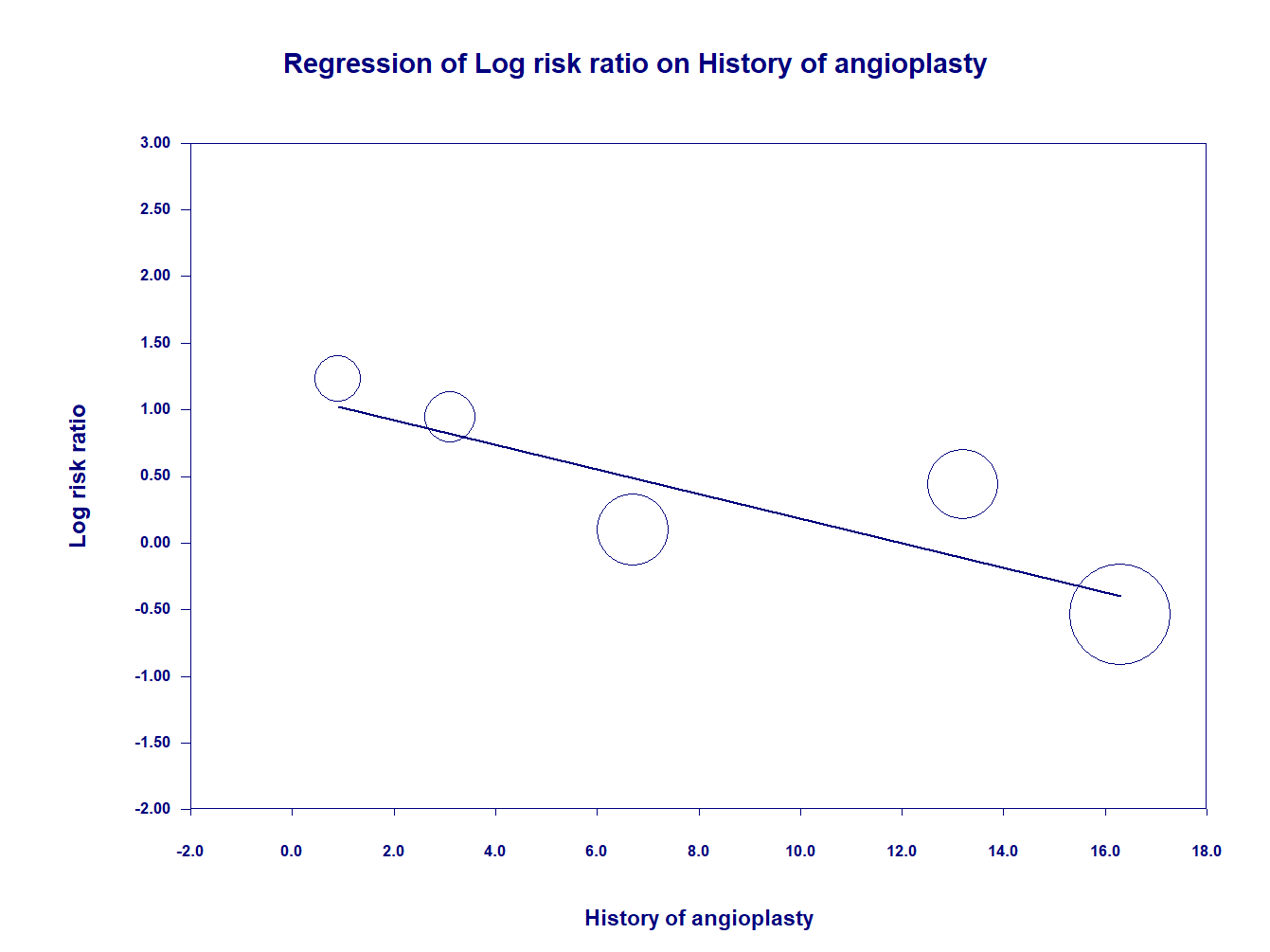
**

**Figure S13. Meta-regression of 1-year mortality and hypertension** (Coeff; -0.045, p=0.0034)

**
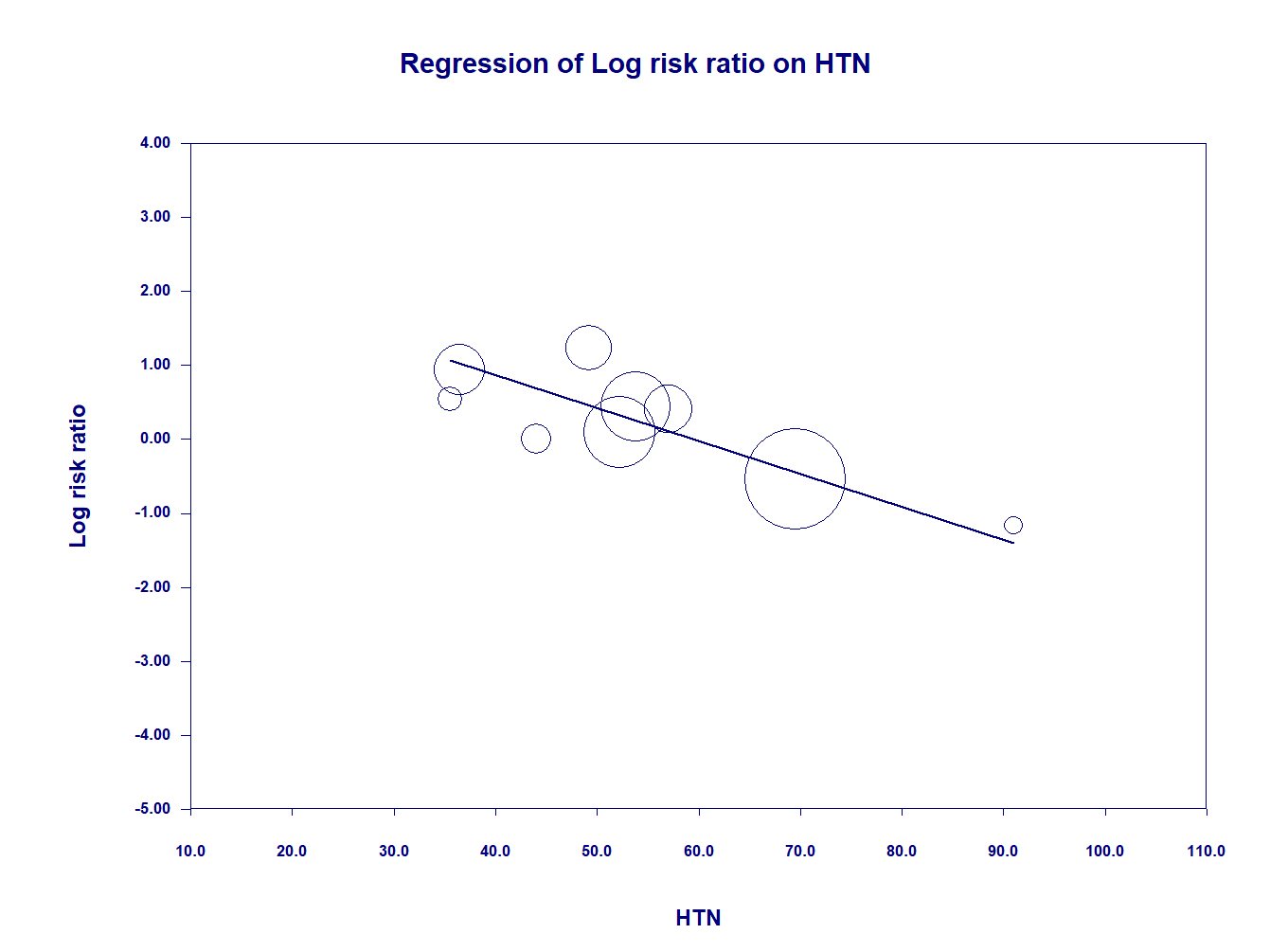
**
